# Efficacy of Platelet-Rich Plasma Augmentation in Anterior Cruciate Ligament Reconstruction: An Updated Systematic Review and Meta-Analysis of Clinical Trials

**DOI:** 10.7759/cureus.94181

**Published:** 2025-10-09

**Authors:** Muhammad Tayyab, Zawar Ahmad, Rizwan Akbar, Mahmood Ahmad, Suleman Shah, Sajida Khan, Iqra Hussain, Ameer Afzal Khan, Rahman Syed, Anfal Khan, Mohsin Ali

**Affiliations:** 1 Trauma and Orthopaedics, Bradford Teaching Hospitals NHS Foundation Trust, Bradford, GBR; 2 Trauma and Orthopaedics, Kettering General Hospital, Kettering, GBR; 3 Trauma and Orthopaedics, University College Hospital, London, GBR; 4 Trauma and Orthopaedics, Milton Keynes University Hospital, Milton Keynes, GBR; 5 Nursing, Fatima College of Health Sciences, Al Ain, ARE; 6 Internal Medicine, Saidu Medical College, Saidu Sharif, PAK

**Keywords:** anterior cruciate ligament reconstruction, graft healing, meta-analysis, platelet-rich plasma, randomized controlled trials

## Abstract

This updated systematic review and meta-analysis evaluated the efficacy and safety of platelet-rich plasma (PRP) augmentation in anterior cruciate ligament reconstruction (ACLR). A comprehensive search of PubMed, the Cochrane Central Register of Controlled Trials, trial registries, and grey literature up to August 13, 2025, identified 16 randomized controlled trials comprising 1,085 participants (545 PRP; 540 control) with follow-up durations ranging from 3 to 24 months. Compared with standard ACLR, PRP significantly reduced pain at three months (mean difference (MD) -0.76; 95% confidence interval (CI) -1.90 to -0.39) and six months (MD -0.67; 95% CI -1.24 to -0.11), but no difference remained at 12 months (MD -0.16; 95% CI -0.51 to 0.18). Functional outcomes demonstrated small, non-significant gains in Lysholm Knee Scoring Scale and International Knee Documentation Committee (IKDC) scores at three and six months and no effect at 12 months, while the Tegner Activity Scale showed a modest but significant improvement at six months (MD 0.46; 95% CI 0.06 to 0.85) that disappeared thereafter. Tunnel widening at 12 months was non-significant for both tibial (MD 0.16; 95% CI -0.46 to 0.78) and femoral tunnels (MD 0.27; 95% CI -0.07 to 0.62). KT-1000 arthrometer (KT-1000) outcomes slightly favored control (MD 0.48; 95% CI 0.12 to 0.83), indicating minimally greater anterior-posterior stability without PRP. The certainty of evidence was moderate to high for pain outcomes and low to very low for functional and structural outcomes due to heterogeneity and small sample sizes. In summary, PRP augmentation provides modest short-term benefits in pain reduction and functional recovery but does not improve long-term stability, graft maturation, or tunnel integrity, and therefore should currently be considered an adjunct rather than a routine practice in ACLR.

## Introduction and background

Anterior cruciate ligament (ACL) rupture is one of the most common knee ligament injuries, especially in young, active athletes. If left untreated, this injury can result in knee instability, discomfort, functional impairment, and an increased risk of secondary meniscal or chondral damage [1]. ACL reconstruction (ACLR) is the gold standard for regaining knee stability and returning to pre-injury activity levels [2]. Despite significant advances in surgical techniques, graft selection, fixation methods, and rehabilitation protocols, a subset of patients continues to have poor outcomes, such as persistent pain, delayed graft maturation, tunnel widening, and incomplete functional recovery, particularly during the first year after surgery [3,4].

Biological augmentation has emerged as a promising technique for enhancing recovery following ACLR. Among the existing treatments, platelet-rich plasma (PRP), an autologous concentration of platelets high in growth factors and cytokines, has drawn attention [5]. PRP is generally indicated in patients undergoing primary ACL reconstruction who are expected to benefit from enhanced graft healing and biological integration, particularly those without systemic contraindications such as coagulopathies or platelet disorders. PRP is believed to enhance angiogenesis, reduce inflammation, and promote graft integration and ligamentization [6]. Several randomised controlled trials (RCTs) have looked at PRP administered intraoperatively to grafts, tunnels, or donor sites, as well as postoperative injections into the knee joint. Patient-reported pain (Visual Analog Scale, or VAS), knee function (Lysholm Knee Scoring Scale, Tegner Activity Scale, and International Knee Documentation Committee (IKDC) scores), objective stability (KT-1000 arthrometer measurements and Lachman test), and imaging markers such as tunnel widening or graft signal intensity are among the outcomes evaluated [7-10].

However, the evidence remains inconsistent. Some meta-analyses have suggested that PRP lowers early postoperative pain and improves short-term functional scores [7,8]; however, others have found no clinically significant improvements for long-term function, stability, or graft maturation [9,10]. These disparities could be attributed to differences in PRP preparation (leukocyte-rich vs. leukocyte-poor; activated vs. non-activated), timing and frequency of delivery (intraoperative vs. postoperative; single vs. several doses), and follow-up times. Furthermore, many previous assessments missed the most recent RCTs and failed to assess the certainty of evidence using formal frameworks such as the Grading of Recommendations Assessment, Development, and Evaluation (GRADE) approach [9-11].

Given these limitations, an updated and complete synthesis of the literature is necessary. The current study aims to evaluate the efficacy of PRP augmentation in ACLR using an updated systematic review and meta-analysis of RCTs. We sought to know whether PRP outperformed traditional ACLR in terms of pain relief, functional recovery, knee stability, tunnel widening, and graft maturation at short-, medium-, and long-term follow-up.

## Review

Methodology

Review Protocol and Registration

This systematic review and meta-analysis were carried out in accordance with the Cochrane Handbook for Systematic Reviews of Interventions and reported using the Preferred Reporting Items for Systematic Reviews and Meta-Analyses (PRISMA) 2020 criteria [12,13]. A preliminary protocol was prospectively registered with the International Prospective Register of Systematic Reviews (PROSPERO) (registration number: CRD420251142319), which defined the objectives, eligibility criteria, outcomes of interest, and statistical methods to reduce bias and increase transparency.

Search Strategy

A comprehensive search of PubMed/MEDLINE and the Cochrane Central Register of Controlled Trials (CENTRAL) was conducted from the database's inception until August 13, 2025, with a focus on papers published in English. The search strategy included both restricted vocabulary and free-text terms such as "platelet-rich plasma," "platelet concentrate," "anterior cruciate ligament reconstruction," "ACLR," and "randomised controlled trial." The search method was created in collaboration with an experienced medical librarian, tested, and refined. To guarantee completeness, we hand-searched the reference lists of the included papers and relevant systematic reviews, as well as trial registries (such as ClinicalTrials.gov) and grey literature (research and reports that are not published through traditional commercial or academic channels). All citations were added to Zotero (Corporation for Digital Scholarship, Fairfax, VA, USA) for management and deduplication [14].

Eligibility Criteria

Studies were eligible for inclusion if they were randomized controlled trials (RCTs) or prospective comparative clinical trials involving participants undergoing primary ACL reconstruction. The intervention had to consist of PRP or other platelet concentrates applied intraoperatively and/or postoperatively, compared with ACL reconstruction performed without PRP. Eligible studies were required to report at least one predefined primary or secondary outcome of interest, such as graft healing, tunnel integration, functional recovery, or complication rates.

We excluded retrospective or non-comparative studies such as case series or case reports, as these designs, while occasionally containing original data, are inherently limited by potential selection bias and lack of control groups, which can compromise the validity of pooled analyses. Additionally, we excluded animal or in vitro studies, reviews, editorials, commentaries, and letters without original comparative data, as well as studies lacking a suitable control group.

Study Selection

All records were screened independently by two reviewers (M.T., A.A.K) in two phases. First, titles and abstracts were screened against inclusion criteria; second, full texts of potentially eligible articles were assessed. Discrepancies were resolved by discussion, and unresolved disagreements were adjudicated by a third reviewer (R.S). A PRISMA 2020 flow diagram documented the selection process, including reasons for exclusion at the full-text stage.

Data Extraction

Data were extracted independently by two reviewers (Z.A., A.K.) using a standardized data-collection form. Extracted information included study characteristics (first author, year, country, study design, sample size), intervention details (PRP preparation, dosage, timing, site of application), comparator details, and follow-up duration. Primary outcomes were graft healing and integration parameters (MRI signal intensity, tunnel healing, graft maturation scores). Secondary outcomes included functional scores (IKDC [15], Lysholm [16], Tegner [17], VAS [18]), knee stability (Lachman [19], pivot shift [19], KT-1000 [20]), tunnel widening, donor-site morbidity, graft re-rupture or revision, adverse events, and return-to-sport time. For continuous outcomes, sample size, mean, and standard deviation were extracted; for dichotomous outcomes, the number of events and total participants were recorded. Any discrepancies were resolved by consensus with a third reviewer.

Risk of Bias and Certainty of Evidence

The methodological quality of included RCTs was appraised using the Cochrane Risk of Bias 2 (RoB 2) tool [21]. The certainty of evidence for each outcome was assessed using the Grading of Recommendations Assessment, Development, and Evaluation (GRADE) approach [22], taking into account risk of bias, inconsistency, indirectness, imprecision, and publication bias. “Summary of Findings” tables were prepared to present pooled effect estimates and certainty ratings for primary and key secondary outcomes.

Statistical Analysis

All statistical analyses were conducted using Review Manager (RevMan) version 5.4 (Nordic Cochrane Centre, Copenhagen, Denmark) [23]. Continuous outcomes (e.g., VAS [18], IKDC [15], Lysholm [16], Tegner scores [17]; tunnel widening) were pooled as mean differences (MDs) or standardised mean differences (SMDs) with 95% confidence intervals (CIs). Dichotomous outcomes (e.g., graft re-rupture, revision, adverse events, return-to-sport) were expressed as risk ratios (RRs) with 95% CI. Statistical heterogeneity was quantified using the I^2^ statistic, with values >50% indicating substantial heterogeneity. Given anticipated clinical variability in PRP preparation, application site, and graft type, a random-effects model was used to generate pooled estimates.

Results

Study Selection and Characteristics

**Figure 1:**
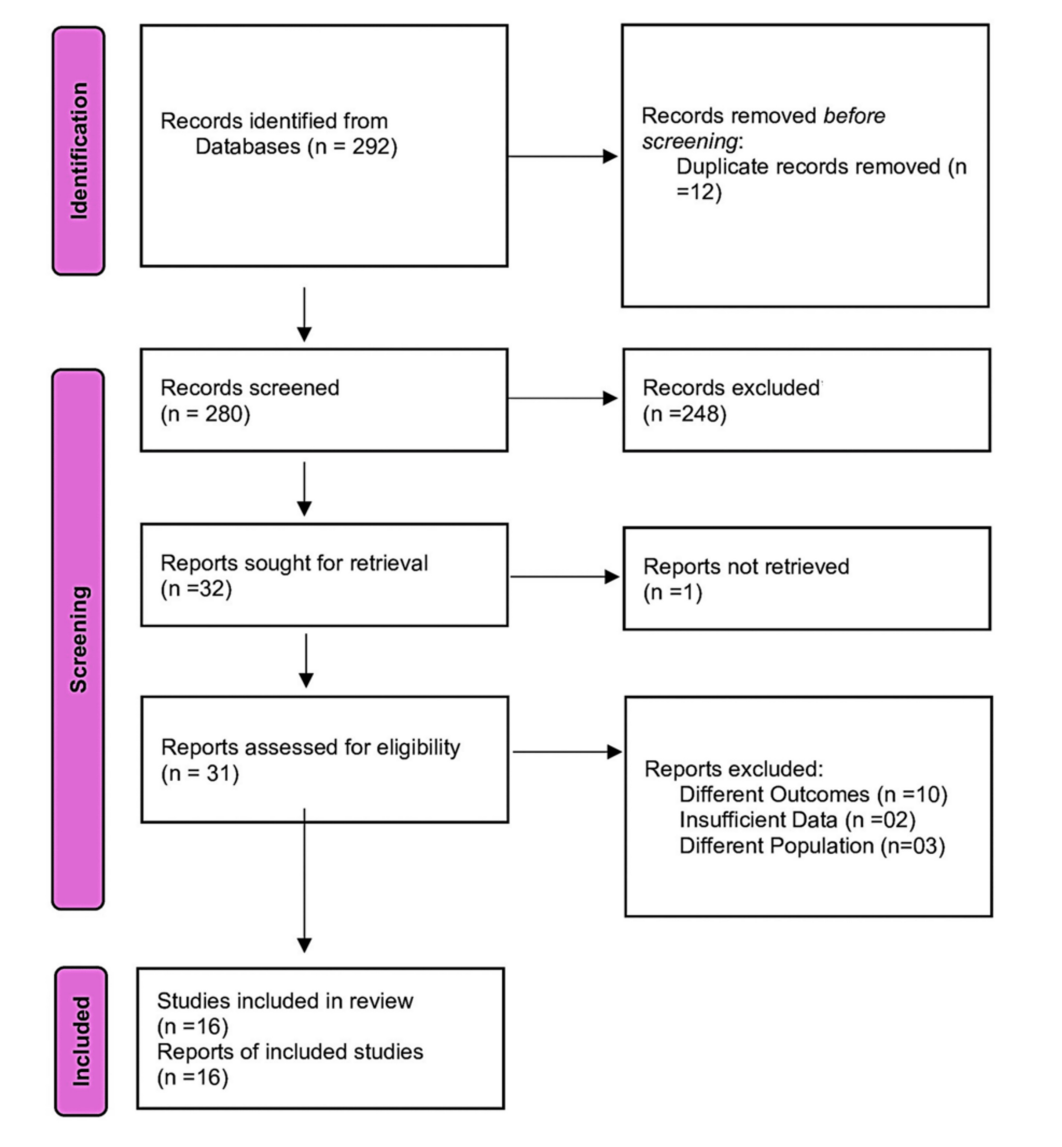
PRISMA Flow Diagram of Study Selection for the Systematic Review and Meta-Analysis PRISMA: Preferred Reporting Items for Systematic Reviews and Meta-Analyses

A total of 16 RCTs [24-39] comprising 1,085 participants were included in this updated systematic review and meta-analysis. Figure 1 shows the study selection PRISMA flow diagram.

All studies compared PRP augmentation with standard ACLR without PRP. The follow-up duration ranged from 3 to 24 months. Most trials evaluated functional outcomes using the Lysholm score [16], IKDC score [15], Tegner Activity Scale [17], and VAS for pain [18], while several assessed tunnel widening (tibial or femoral) as a surrogate for graft healing and fixation. The PRP protocols varied across studies, with some using platelet-rich fibrin (PRF) or platelet-rich growth factors (PRGF). Table 1 summarizes the characteristics of the included studies.

**Table 1:** Characteristics of Included Studies RCT: randomized controlled trial; I: intervention group; C: control group; PRP: platelet-rich plasma; PR: platelet-rich; PRF: platelet-rich fibrin; PRGF: plasma rich growth factors; KOOS: Knee Injury and Osteoarthritis Outcome Score; VAS: Visual Analog Scale; IKDC: International Knee Documentation Committee; VISA: Victorian Institute of Sport Assessment; ADT: anterior drawer test; KT-1000: KT-1000 arthrometer; NR: not reported

First Author (Year)	Study Design	Sample Size	Intervention/Control	Follow-Up	Outcomes
Kumar (2022) [24]	RCT	I=35, C=35	PRP/Non-PRP	12 months	Post-op 6 and 12 week Lachman ADT and Lysholm score
Sözkesen (2018) [25]	RCT	I=18, C=26	PRP/Non-PRP	3 months	Tibial Tunnel + Lysholm score, Tegner Activity Scale, and a KT-1000 arthrometer device
Starantzis (2014) [26]	RCT	I=25, C=26	PRP/Non-PRP	12 months	Mean tunnel diameter + Lysholm + Rolimeter
Ye (2024) [27]	RCT	I=60, C=60	PRP/Conventional	12 months	KOOS, VAS, IKDC, Tegner, Lysholm
Gong (2022) [28]	RCT	I=30, C=30	PRP/Non-PRP	12 months	VAS, Lysholm score, Tegner score, 2000IKDC, Femoral tunnel, Tibial tunnel
Nin (2009) [29]	RCT	I=50, C=50	PR Gel	12 months	VAS, KT 1000, IKDC
Walters (2018) [30]	RCT	I=27, C=23	PRP	24 months	VAS, IKDC
Cervillin (2012) [31]	RCT	I=20, C=20	PRP	12 months	VAS, VISA scores
de Almeida (2012) [32]	RCT	I=12, C=15	PRP	6 months	Gap area, VAS, Lysholm, IKDC, Tegner
Mirzatolooei (2013) [33]	RCT	I=23, C=23	PRP	3 months	Femoral and tibial tunnel widening
Vadalà (2013) [34]	RCT	I=20, C=20	PRP	12 months	VAS, IKDC, Lysholm score, KT-1000 tibial and femoral tunnel diameter
Lin (2024) [35]	RCT	I=8, C=10	PRP	11-12 months	IKDC, Lysholm, tibial and femoral tunnel diameter
Seijas (2016) [36]	RCT	I=23, C=20	PRGF	24 months	VAS score
Ventura (2005) [37]	RCT	I=10, C=10	PRF	NR	KT-1000, KOOS, Tegner score
Ji (2017) [38]	RCT	I=17, C=19	PRP	12 months	VAS, IKDC, Lysholm score
Munde (2023) [39]	RCT	I=43, C=44	PRP	6 months	Graft maturity, graft intensity, Figueroa score

Pain Outcomes (VAS)

**Figure 2:**
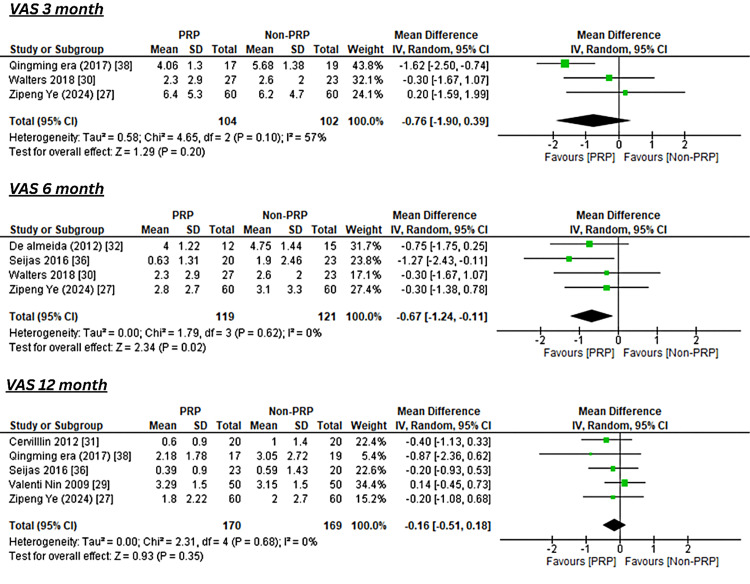
Forest Plot of Pooled Mean Differences in VAS Pain Scores at 3, 6, and 12 Months After ACL Reconstruction With or Without PRP Augmentation References [38,30,27,32,36,31,29] VAS: Visual Analog Scale; ACL: anterior cruciate ligament; PRP: platelet-rich plasma

At three months, pooled data from three studies demonstrated a significant reduction in pain favoring PRP (MD -0.76; 95% CI -1.90 to -0.39; I^2^=57%). At six months, four studies also favored PRP with a smaller but significant reduction in pain (MD -0.67; 95% CI -1.24 to -0.11; I^2^=0%). By 12 months, pooled data from five studies showed no significant difference between PRP and control (MD -0.16; 95% CI -0.51 to 0.18; I^2^=0%) as shown in Figure 2.

Tunnel Widening

**Figure 3:**
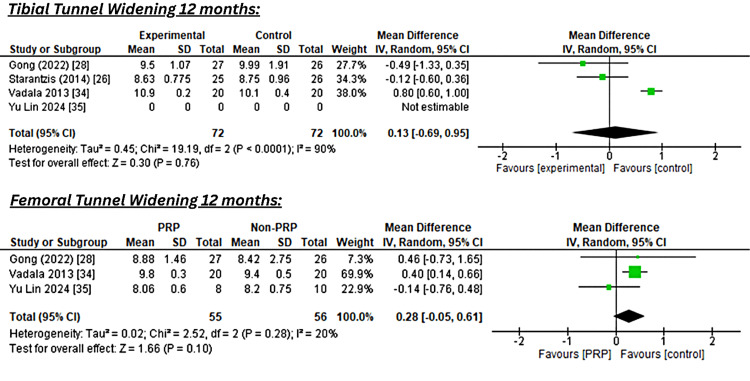
Forest Plot of Pooled Mean Differences in Tibial and Femoral Tunnel Widening at 12 Months After ACL Reconstruction With or Without PRP Augmentation References [28,26,34,35] ACL: anterior cruciate ligament; PRP: platelet-rich plasma

Pooled analysis of tibial tunnel widening at 12 months from four studies (n=144) showed a non-significant difference favoring the control group (MD 0.16; 95% CI -0.46 to 0.78; I^2^=86%). Femoral tunnel widening at 12 months from three studies showed a similar pattern (MD 0.27; 95% CI -0.07 to 0.62; I^2^=23%), as shown in Figure 3.

Functional Outcomes

Lysholm score: Six studies at three months (n=344) showed a non-significant improvement favoring PRP (MD 0.75; 95% CI -2.92 to 4.43; I^2^=51%). At six months, three studies (n=278) found a similar small effect (MD 2.83; 95% CI -0.96 to 6.62; I^2^=55%). At 12 months, five studies (n=294) revealed no difference (MD 0.04; 95% CI -2.04 to 2.13; I^2^=0%), as shown in Table 2.

**Table 2:** Summary of Pooled Effect Estimates From Forest Plots for the Respective Outcomes PRP: platelet-rich plasma; ACL: anterior cruciate ligament; MD: mean difference; CI: confidence interval; I^2^: heterogeneity statistic; IKDC: International Knee Documentation Committee; MCID: minimal clinically important difference; NS: not significant; post-op: postoperative

Outcome	Timepoint	Studies (n)	Pooled MD (95% CI)	I^2^	Favored Group	Clinical Importance
Lysholm Score [16]	3 months	6	0.75 (-2.92, 4.43)	51%	PRP	Trivial (below MCID ~8-10)
	6 months	4	2.83 (-0.96, 6.62)	55%	PRP	Trivial
	12 months	5	0.04 (-2.04, 2.13)	0%	No difference	Trivial
Tegner Activity Score [17]	3 months	3	0.00 (-0.43, 0.44)	29%	No difference	Trivial (MCID ~1)
	6 months	3	0.46 (0.06, 0.85)	1%	PRP	Trivial (below MCID ~1)
	12 months	2	-1.51 (-4.83, 1.80)	0%	PRP (but NS)	Trivial
IKDC Score [15]	3 months	3	1.03 (-5.83, 7.90)	75%	PRP	Trivial (MCID ~11-13)
	6 months	3	1.39 (-1.79, 4.57)	0%	PRP	Trivial
	12 months	5	0.98 (-1.30, 3.27)	12%	PRP	Trivial
KT-1000 [20]	Post-op	4	0.48 (CI 95%: 0.12-0.83)	0%	Non-PRP	Minimally important clinically

Tegner Activity Scale: At three months, three studies (n=220) found no difference between groups (MD 0.00; 95% CI -0.43 to 0.44; I^2^=29%). At six months, three studies (n=198) showed a small but statistically significant benefit of PRP (MD 0.46; 95% CI 0.06 to 0.85; I^2^=1%). At 12 months, two studies (n=167) demonstrated a non-significant effect (MD -1.51; 95% CI -4.83 to 1.80; I^2^=0%), as shown in Table 2.

IKDC score: Across three studies at three months (n=212), the pooled effect favored PRP but was not significant (MD 1.03; 95% CI -5.83 to 7.90; I^2^=75%). At six months (n=198), MD 1.39 (95% CI -1.79 to 4.57; I^2^=0%) favored PRP without reaching statistical significance. At 12 months (n=293), MD 0.98 (95% CI -1.30 to 3.27; I^2^=12%) showed no significant difference, as shown in Table 2.

KT-1000 arthrometer: Four studies reported KT-1000 outcomes, which favored the control (non-PRP) group (MD 0.48; 95% CI 0.12 to 0.83; I^2^=0%). This difference may reflect slightly better anterior-posterior knee stability in the non-PRP group, although the effect was minimally clinically important, as shown in Table 2.

Certainty of Evidence

GRADE assessments indicated moderate to high certainty evidence for VAS outcomes and low to very low certainty for functional scores and tunnel widening due to heterogeneity, small sample sizes, and imprecision of effect estimates, as shown in Table 3.

**Table 3:** The Summary of Grade Assessment * The risk in the intervention group (and its 95% CI) is based on the assumed risk in the comparison group and the relative effect of the intervention (and its 95% CI). GRADE Working Group grades of evidence: High certainty: We are very confident that the true effect lies close to that of the estimate of the effect. Moderate certainty: We are moderately confident in the effect estimate: the true effect is likely to be close to the estimate of the effect, but there is a possibility that it is substantially different. Low certainty: Our confidence in the effect estimate is limited: the true effect may be substantially different from the estimate of the effect. Very low certainty: We have very little confidence in the effect estimate: the true effect is likely to be substantially different from the estimate of effect. CI: confidence interval; MD: mean difference; GRADE: Grading of Recommendations Assessment, Development, and Evaluation; VAS: Visual Analog Scale; IKDC: International Knee Documentation Committee; RCT: randomised controlled trial; PRP: platelet-rich plasma; ACL: anterior cruciate ligament

Patient or Population: ACL Reconstruction Setting: Intervention: PRP Comparison: Control					
Outcomes	Anticipated Absolute Effects* (95% CI)		Relative Effect (95% CI)	No. of Participants (Studies)	Certainty of the Evidence (GRADE)
	Risk With Control	Risk With PRP			
KT-1000	The mean KT-1000 was 0	MD 0.48 higher (0.12 higher to 0.83 higher)	-	204 (4 RCTs)	⨁⨁⨁⨁ High
VAS 3 months	The mean VAS 3 months was 0	MD 0.76 lower (1.9 lower to 0.39 higher)	-	206 (3 RCTs)	⨁⨁◯◯ Low
VAS 6 months	The mean VAS 6 months was 0	MD 0.67 lower (1.24 lower to 0.11 lower)	-	240 (4 RCTs)	⨁⨁⨁◯ Moderate
VAS 12 months	The mean VAS 12 months was 0	MD 0.16 lower (0.51 lower to 0.18 higher)	-	339 (5 RCTs)	⨁⨁⨁⨁ High
Lysholm 3 months	The mean Lysholm 3 months was 0	MD 0.75 higher (2.92 lower to 4.43 higher)	-	344 (6 RCTs)	⨁⨁◯◯ Low
Lysholm 6 months	The mean Lysholm 6 months was 0	2.83 (-0.96, 6.62)	-	278 (3 RCTs)	⨁⨁⨁◯ Moderate
Lysholm Score 12 months	The mean Lysholm Score 12 months was 0	MD 0.04 higher (2.04 lower to 2.13 higher)	-	294 (5 RCTs)	⨁⨁⨁◯ Moderate
Tegner Score 3 months	The mean Tegner Score 3 months was 0	MD 0 (0.43 lower to 0.44 higher)	-	220 (3 RCTs)	⨁⨁⨁◯ Moderate
Tegner Scale 6 month	The mean Tegner Scale 6 months was 0	MD 0.46 higher (0.06 higher to 0.85 higher)	-	198 (3 RCTs)	⨁⨁⨁⨁ High
12 months Tegner Score	The mean 12 months Tegner Score was 0	MD 1.51 lower (4.83 lower to 1.8 higher)	-	167 (2 RCTs)	⨁⨁⨁◯ Moderate
IKDC 3 months	The mean IKDC 3 months was 0	MD 1.03 higher (5.83 lower to 7.9 higher)	-	212 (3 RCTs)	⨁◯◯◯ Very low
IKDC 6 months	The mean IKDC 6 months was 0	MD 1.39 higher (1.79 lower to 4.57 higher)	-	198 (3 RCTs)	⨁⨁◯◯ Low
IKDC 12 months	The mean IKDC 12 months was 0	MD 0.98 higher (1.3 lower to 3.27 higher)	-	293 (5 RCTs)	⨁⨁◯◯ Low
Tibial Tunnel Widening 12 months	The mean tibial Tunnel Widening 12 months was 0	MD 0.13 higher (0.69 lower to 0.95 higher)	-	144 (4 RCTs)	⨁◯◯◯ Very low

Discussion

This updated systematic review and meta-analysis, which includes 16 RCTs and a total sample size of 1,085 patients, is the most comprehensive synthesis to date on the effect of PRP augmentation in ACLR. The study found that PRP had moderate but statistically significant short- and mid-term advantages, notably in terms of lowering postoperative pain and increasing functional outcomes as determined by instruments including the IKDC [15], Lysholm [16], and Tegner [17] activity ratings. However, these benefits did not last beyond 12 months, with no consistent improvements in pain, functional recovery, graft maturation, or structural characteristics such as tunnel widening. Importantly, objective assessments of knee stability (e.g., KT-1000 arthrometer [20], Lachman test [19], pivot shift [19]) and graft healing as measured by MRI indicated no clinically significant differences between the PRP-augmented and regular ACLR groups.

Comparison With Prior Evidence

The findings of this meta-analysis are consistent with and expand on earlier research in this area. Zhu et al. [8] previously showed that PRP improved pain and functional results in the early postoperative period but did not give long-term clinical or radiological advantages. Similarly, in a recent randomised clinical trial, Ye et al. [27] discovered that while intra-articular PRP injections improved graft maturity at early time points, they did not result in better patient-reported outcomes or objective stability at 12 months. Another systematic review by de Andrade et al. [40] supported these findings, stating that while PRP showed modest improvements in subjective outcomes such as VAS and Lysholm scores, there were no benefits in objective measures such as tunnel widening, graft ligamentization, or knee laxity.

Other RCTs have provided additional detail about the potential involvement of PRP. Munde et al. [39] found that PRP considerably improved graft healing and Lysholm scores [16] after six months, indicating that early biological effects can result in measurable clinical changes. Seijas et al. [36] found MRI evidence of faster patellar tendon graft remodelling with PRP supplementation at four and six months, but these differences disappeared by 12 months. Collectively, these findings highlight the temporary nature of PRP's effect: it is favourable in the early healing period but insufficient to change long-term outcomes, which are more dependent on mechanical stability, surgical technique, and rehabilitation adherence.

A more recent meta-analysis [9] examined the impact of PRP analogues over a year and concluded that while IKDC scores [15] improved slightly, they did not exceed minimal clinically important difference (MCID) thresholds, and there were no significant differences in Lysholm [16] or Tegner [17] activity scores. This highlights the fact that, even when statistically significant, the therapeutic usefulness of PRP's advantages can be restricted.

Biological Rationale

PRP is a biologically active concentrate rich in platelets, growth factors (e.g., platelet-derived growth factor (PDGF), vascular endothelial growth factor (VEGF), and transforming growth factor-beta (TGF-β)), and cytokines that promote angiogenesis, collagen deposition, and fibroblast proliferation. In theory, this could speed up graft ligamentization and tendon-bone integration after ACLR. PRP treatment has been shown in experimental and histological studies to increase cellular proliferation and early collagen fibre alignment [41,42]. Clinically, this could explain the reported decrease in postoperative discomfort and early functional gains. However, the short half-life of growth factors, heterogeneity in PRP composition, and the fast resolution of the inflammatory phase may limit the long-term effects. Mechanical and biological mechanisms such as graft remodelling, tunnel widening, and neuromuscular adaptation eventually eclipse PRP's temporary biologic effects.

Heterogeneity Across Studies

One of the major challenges in interpreting the evidence is the substantial heterogeneity among trials. PRP preparation methods differ significantly, particularly in platelet concentration, leukocyte presence (leukocyte-rich vs. leukocyte-poor PRP), and activation protocols. The site and time of treatment also vary: some studies injected PRP intraoperatively into the graft or tunnels, while others employed postoperative intra-articular injections. Furthermore, variations in graft type (hamstring autograft vs. bone-patellar tendon-bone), rehabilitation regimes, and surgical procedures may all influence outcomes. This heterogeneity most likely explains some of the observed effects' inconsistencies and emphasises the importance of standardisation in PRP research.

Clinical Implications

From a clinical aspect, PRP augmentation in ACLR may be most beneficial for patients seeking rapid early recovery, with a focus on pain reduction and functional gains during the critical first three to six months of rehabilitation. However, in the absence of significant long-term gains, PRP should not be advised as a routine adjunct in ACLR. Cost, resource utilisation, and procedural complexity must all be considered; the modest and short-term benefits may not be sufficient to warrant widespread adoption, especially in healthcare systems where cost-effectiveness is crucial.

Limitations

This study has significant limitations, which are consistent with those found in the included trials. First, despite combining 16 RCTs, several outcomes were informed by only a small fraction of studies, reducing statistical power. Second, most trials had relatively short follow-up periods, rarely reaching 12 months, leaving long-term graft survival, re-rupture rates, and return-to-sport outcomes unexplored. Third, insufficient reporting of PRP methods restricted subgroup analysis, making it impossible to draw firm conclusions about the relative efficacy of leukocyte-rich vs. leukocyte-poor PRP or graft vs. tunnel vs. intra-articular administration. Fourth, while funnel plots and Egger's test were utilised where possible, the small number of studies per outcome hampered the assessment of publication bias. Finally, functional scores such as IKDC [15] and Lysholm [16], though validated, may lack sensitivity to detect subtle but clinically relevant differences in sports performance or patient satisfaction.

Future Research Directions

Future trials should follow standardised PRP preparation and application techniques to reduce heterogeneity and improve comparability. Long-term follow-up (≥24-36 months) is crucial for assessing outcomes, including graft re-rupture, revision rates, donor-site morbidity, and sustained functional recovery. To correlate biological repair with functional results, studies should use both patient-reported outcomes and advanced imaging modalities such as quantitative MRI. Furthermore, established MCIDs should be employed to distinguish between statistically and clinically significant changes. Finally, cost-effectiveness assessments are required to inform healthcare decision-making, particularly in resource-constrained settings.

## Conclusions

This meta-analysis reveals that while PRP augmentation in ACLR gives moderate short-term benefits in pain alleviation and functional results, it does not consistently improve stability, graft maturation, or tunnel integrity. Although biologically reasonable, PRP's clinical value is restricted by temporary effects and methodological variation. PRP should currently be regarded as an adjunctive alternative rather than routine therapy in ACLR, pending additional high-quality evidence from large, standardised, long-term randomised trials.
